# The role of phenolic OH groups of flavonoid compounds with H-bond formation ability to suppress amyloid mature fibrils by destabilizing β-sheet conformation of monomeric Aβ17-42

**DOI:** 10.1371/journal.pone.0199541

**Published:** 2018-06-28

**Authors:** Sahar Andarzi Gargari, Abolfazl Barzegar, Alireza Tarinejad

**Affiliations:** 1 Department of Biotechnology, Faculty of Agriculture, Azarbaijan Shahid Madani University, Tabriz, Iran; 2 Student Research Committee, Tabriz University of Medical Sciences, Tabriz, Iran; 3 Department of Biophysics, Research Institute for Fundamental Sciences (RIFS), University of Tabriz, Tabriz, Iran; 4 Department of Medical Biotechnology, Faculty of Advanced Medical Sciences, Tabriz University of Medical Sciences, Tabriz, Iran; Bose Institute, INDIA

## Abstract

Alzheimer’s disease (AD) is a kind of brain disease that arises due to the aggregation and fibrillation of amyloid β-peptides (Aβ). The peptide Aβ17–42 forms U-shape protofilaments of amyloid mature fibrils by cross-β strands, detected in brain cells of individuals with AD. Targeting the structure of Aβ17–42 and destabilizing its β-strands by natural compounds could be effective in the treatment of AD patients. Therefore, the interaction features of monomeric U-shape Aβ17–42 with natural flavonoids including myricetin, morin and flavone at different mole ratios were comprehensively studied to recognize the mechanism of Aβ monomer instability using molecular dynamics (MD) simulations. We found that all flavonoids have tendency to interact and destabilize Aβ peptide structure with mole ratio-dependent effects. The interaction free energies of myricetin (with 6 OHs) and morin (with 5 OHs) were more negative compared to flavone, although the total binding energies of all flavonoids are favorable and negative. Myricetin, morin and flavone penetrated into the core of the Aβ17–42 and formed self-clusters of Aβ17-42-flavonoid complexes. Analysis of Aβ17-42-flavonoids interactions identified that the hydrophobic interactions related to SASA-dependent energy are weak in all complexes. However, the intermolecular H-bonds are a main binding factor for shifting U-shape rod-like state of Aβ17–42 to globular-like disordered state. Myricetin and morin polyphenols form H-bonds with both peptide’s carbonyl and amine groups whereas flavone makes H-bonds only with amine substitution. As a result, polyphenols are more efficient in destabilizing β-sheet structures of peptide. Accordingly, the natural polyphenolic flavonoids are useful in forming stable Aβ17-42-flavonoid clusters to inhibit Aβ17–42 aggregation and these compounds could be an effective candidate for therapeutically targeting U-shape protofilaments’ monomer in amyloid mature fibrils.

## Introduction

Protein misfolding and aggregation is one of the most concerning problems in applied biophysics [1, 2] and molecular medicine [3]. Several human brain diseases occur due to the protein misfolding and aggregation considered as hallmark pathognomonic features of various neurodegenerative diseases, including Alzheimer, Parkinson, Huntington and Prion diseases [4, 5]. Alzheimer’s disease (AD) is a progressive memory loss causes destruction and death of brain cells. Unfortunately, despite various attempts to combat the disease, an effective therapeutic intercession is lacking for this common cause of mortality in the world [6]. The disease is estimated to afflict upward 5.2 million Americans and more than 25 million individuals worldwide [7]. Although the underlying cause of AD is not completely clear, it is apparent that the major pathological features are because of the presence of macroscopic structures aggregation and deposition of β-amyloid peptides (Aβ). The aggregated peptides are derived from amyloid precursor protein by sequential cleaving of β- and γ –secretases [8]. γ-secretases produce the multiple alloforms of peptides such as Aβ40 and Aβ42, with 40 and 42 amino acid residues respectively. Aβ40 peptide is the most abundant form, but in the disease state the level of Aβ42 is raised up and more readily formed aggregates in solution [9]. The aggregation of Aβ42 peptides is necessary in the progression of AD associated with amyloid fibrils [2]. In addition, the major component of brains plaques in AD, are amino-terminal truncated Aβ17–42 peptide corresponded to the amyloid mature fibrils [10, 11].

**Fig 1:**
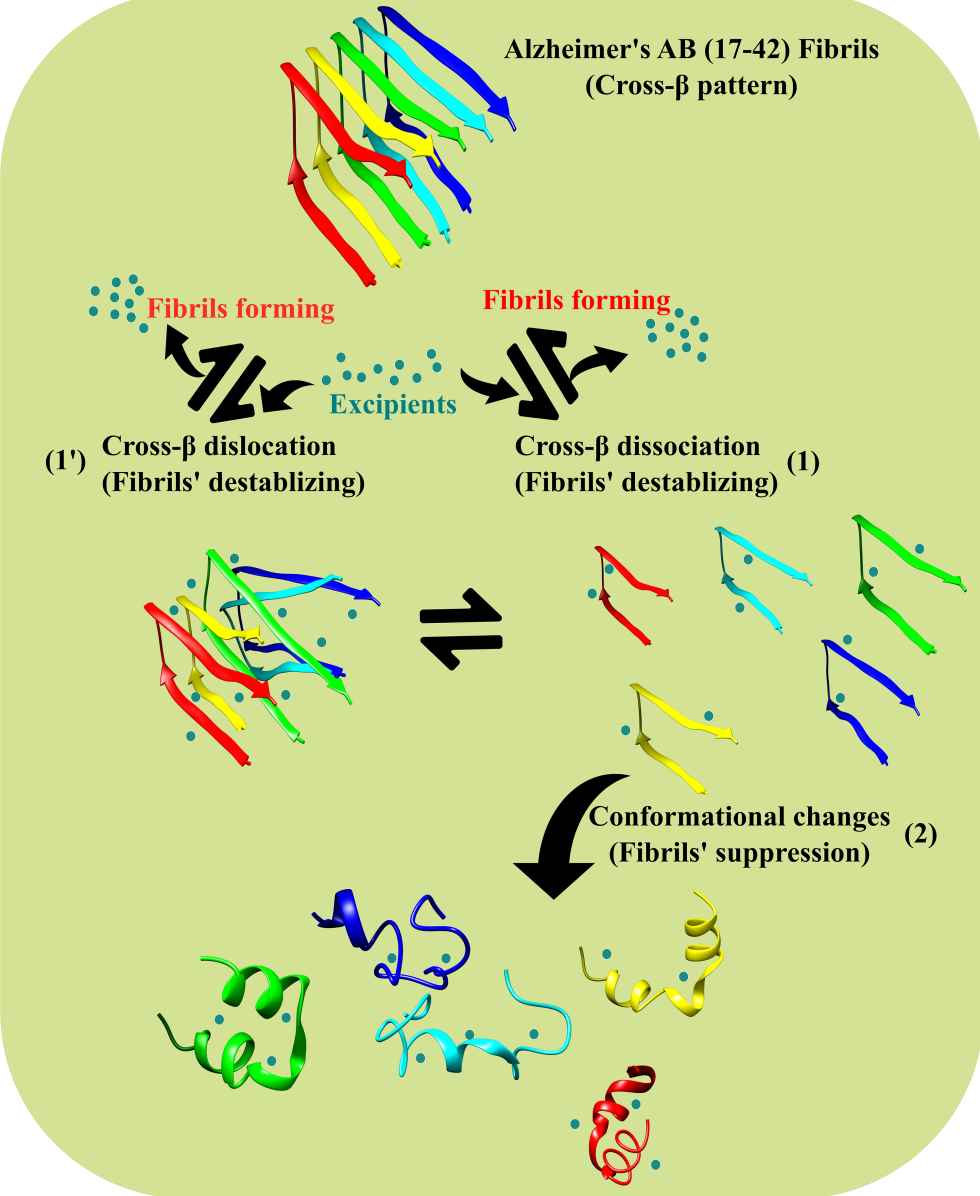
The possible pathways from interacting different excipients with assembled Aβ17–42 peptides of Alzheimer’s cross-β fibrils. Fibrils destabilizing is accompanying with reversible cross-β strands changing (pathways **1** and **1'**). The excipients interact with assembled cross-β fibrils of Aβ17–42 peptides and reversibly destroys the fibril structure of the peptide that can “retard” late stage of amyloid plaques. Pathway **2** is based on the almost irreversible fibrils suppression by converting β-sheet conformations. Reversible destroyed assembled cross-β fibrils are accompanying irreversible β-sheet conformational changing of the Aβ17–42 peptide that can result in crush and suppress late stage of amyloid plaques.

Efforts to design new compounds based on γ-secretase’s inhibitors have met with modest success, since the activities of secretases are vital for normal neuronal function and inhibiting could strengthen the symptoms of AD [12]. Recent efforts are focused on the numerous possibilities that have been directly explored in targeting Aβ peptides using natural compounds [13, 14]. The inhibition of Aβ peptides aggregation [15, 16] and destabilizing amyloid mature fibrils by disturbing cross-β strands [17, 18] are promising therapeutic strategies for progression in early and late stages of AD, respectively. The characterized structural motif in amyloid fibrils is known as cross-β [19]. The cross-β strands are defined the strands of a β-sheet run vertically to the axis of fibril forming mature fibrils [20]. As schematically shown in Fig 1, there are two main strategies to destabilize and/or dissipate the amyloid mature fibrils including; a) changing cross-β strands [17, 21–23] mainly based on alteration of Aβ peptides’ quaternary structure (Fig 1, pathways **1** and **1'**), and b) focusing on the dissociated β strands from fibrils and converting β-sheet conformations to other secondary structures (changing tertiary and predominantly secondary structures of peptides, pathway **2**). The disruption of mature fibrils is an effective procedure to decrease the toxicity of amyloid’s plaques at therapeutic mediation in the late phase of AD. Natural compounds, such as flavonoids may be useful in inhibiting Aβ aggregation and destabilizing preformed fibrils because of their essential bioavailability and low toxicity at therapeutically suitable levels [14]. Various investigations have been conducted on the disruption of mature amyloid fibrils’s cross-β strands and reduction of the toxicity of amyloid’s plaques in the living cells by natural polyphenolic compounds [24, 25]. *In vitro* evidences have proposed that polyphenolic antioxidants are useful as anti-aggregation substances in targeting Aβ peptides [14, 18]. Furthermore *in vivo* studies showed that oral administration of grape derived polyphenolic extracts decrease amyloid plaque and can improve memory and cognitive ability [26, 27]. Molecular dynamics (MD) simulations have been used here for studying atomic details of Aβ peptide interaction with natural excipients [15]. Based on the MD simulation data, Lemkul et al. [17] revealed that the flavonoid morin can bind to the ends of the fibrils and preclude the attachment of an incoming peptide, followed by the reduction of the polymerization rate. The effects of various amyloid aggregation inhibitors on different segments of Aβ peptides have been widely examined by MD simulations [15, 17, 28].

Both experimental and theoretical literatures currently suggest that flavonoids-related compounds inhibit early stage of Aβ aggregation which also disrupt the late stages of the of Alzheimer’s cross-β fibrils, providing the motivation for the present study. The structural feature of oligomeric protofilament state of Aβ17–42 peptides and destabilizing cross oligomeric-β by the interaction with flavonoids (pathways **1** and **1'**) have been extensively studied using MD simulation methods [17, 21, 23, 29]. Besides, despite of wide studies on Aβ peptides’ fibrillogenesis, the conformational stability and affinity of the fibril Aβ17–42 peptide in the case of interaction with excipients remains unclear. In this study, hypothesizing that an excipient interaction with Aβ17–42 peptide (monomeric strands of cross-β protofilaments) destroys the fibril’s formation and suppresses the late stage of amyloid plaques (suppression of late stage plaques, pathways **2**). We aimed to investigate the effectiveness of three different flavonoids including myricetin (with 6 OHs), morin (with 5 OHs) and flavone (with no OH) in the interaction and destabilization effects on the conformation of monomeric cross-β element of Aβ17–42, using MD simulations method.

## Materials and methods

### Aβ peptides setup

Aβ17–42 peptide works as a suitable model of fibrils and is similar to model systems that previously applied for MD simulation studies [17]. The highly hydrophobic monomer of Aβ17–42 peptide would rapidly evolve into fibrils related to amyloid’s plaques with no soluble intermediate forms [30]. We believe the monomeric structural model of Aβ17–42 peptide is the best representative of the fibrils’ cross-β strands conformations of late stage plaques, pathways **2**, in order to study the flavonoids interaction. Therefore, the monomeric subunit of Aβ17–42 fibril, a protofilament structure determined by solid-state NMR (PDB code 2BEG) [31] was applied for MD simulations. To have an uncharged N-termini, amino terminus Leu17 residue of Aβ17–42 peptide was acetylated before any MD simulations.

### Ligands geometries optimizing

**Fig 2:**
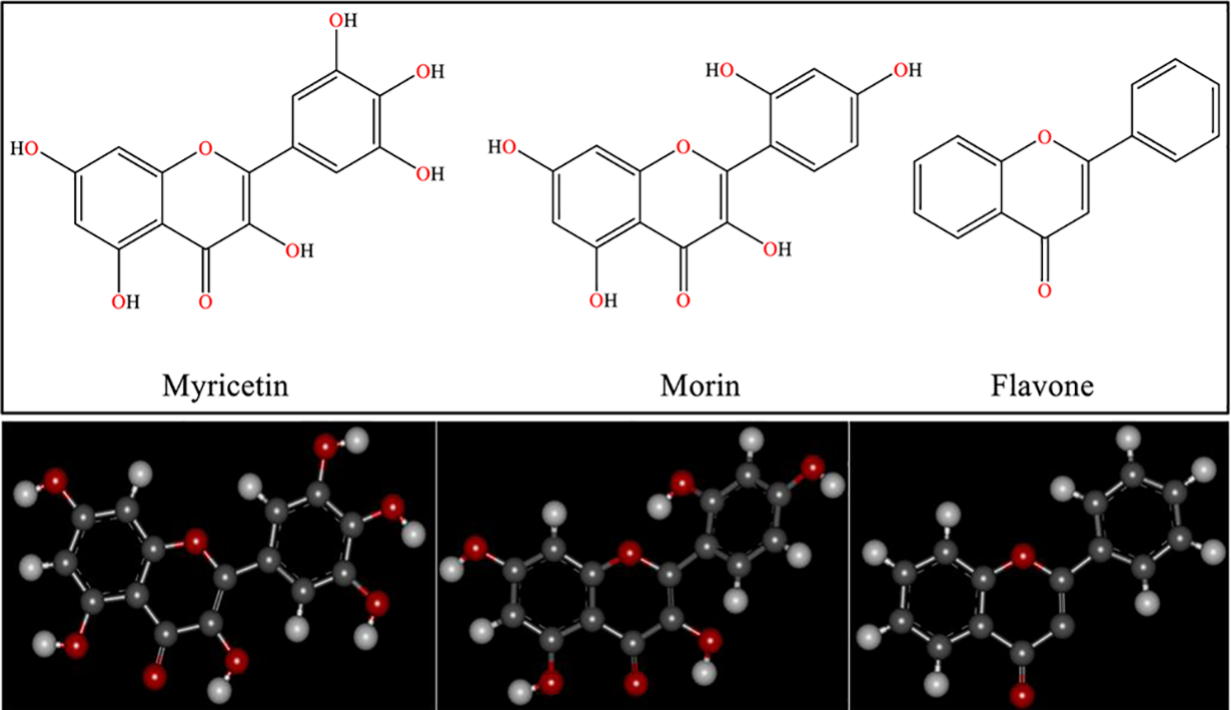
2-D and 3-D (DFT optimized) structures of Myricetin; with 6 functional hydroxyl groups, Morin; with 5 functional hydroxyl groups and Flavone; with no hydroxyl groups substitution.

The initial geometries of different flavonoids including myricetin, morin and flavone were generated by Hyperchem 7 software. The structures were pre-optimized using molecular mechanic MM^+^ force field. Then the geometries optimization was obtained with the semi-empirical AM1 followed by ab initio DFT method with Becke's three-parameter hybrid functional (B3LYP) level using 6–311+G (d,p) basis set. Optimized conformations of myricetin, morin and flavone were provided in Fig 2. The optimized structures were presented into PRODRG 2 server [32] without any coordinate changing to achieve ligands’ topology. Some of the main parameters in topology files such as partial charges were handled based on DFT optimized data. Therefore, the refined topology of ligands from PRODRG 2 server was applied for using MD simulations.

### Simulations setup

A number of 13 diverse systems were prepared and 100 ns simulations of each system have been conducted using GROMACS 5.0.4 package. The details for each system were explained in Table 1. All simulations were conducted using GROMOS96 53A6 force field [33] by applying SPC water model [34]. The systems were separately solvated in explicit water molecules that extend up to 10 Å from any edge of the cubic box to the solute atoms. Previously, different ligands with 10:1 mole ratio were examined to test their anti-aggregation properties on Aβ42 using MD simulation method, such as EGCG peptide [35], naproxen [36], ibuprofen [37] and morin [15]. For theoretical examination of any concentration dependent effects of different flavonoids with Aβ17–42 interactions, wide ranges of mole ratios were applied for flavonoids/Aβ17–42 complexes including 1:1, 2:1, 6:1 and 10:1. The model containing 2:1 mole ratio of flavonoids/Aβ17–42 is almost similar to *in vitro* conditions. For example, 2:1 mol ratio of morin/Aβ was used as the highest concentration of morin to explore anti-amyloidogenic and fibril destabilizing effects of polyphenols *in vitro* [18]. Also, theoretical morin-treated systems containing 2:1 mol ratio of morin/Aβ42 showed that morin inhibits β-peptide aggregation by altering tertiary interactions [17]. Therefore, we have applied different mole ratios of flavonoids/Aβ17–42 from unity to 10 fold higher that could help to examine any possible concentration dependent effects of flavonoid-Aβ interactions. The simulation systems consist of Aβ and Aβ complexes with myricetin, morin and flavone molecules. All systems were neutralized by NaCl counterions. The concentration of NaCl in all simulations systems was 100 mM, by adding appropriate number of Na^+^ and Cl^-^ ions. In all cases, short-range nonbonded interactions were truncated at 1.2 nm, applying long-range dispersion correction to the energy and pressure terms to account for truncation of the van der Waals interactions. The Particle Mesh Ewald (PME) method was utilized for the calculations of long range electrostatic interactions. The LINCS algorithm [38, 39], was used for all bond constraints, allowing an integration time step of 2 fs. Periodic boundary conditions were applied in all directions. The temperature of the systems was preserved at 300 K by using Berendsen weak coupling method and pressure was maintained at 1 bar by utilizing Parrinello-Rahman barostat in constant pressure ensemble. All systems were energy-minimized using the steepest descent method. The minimized systems were equilibrated under NVT (constant volume) and NPT (constant pressure) ensemble conditions, for time scale of 200 ps. Visual Molecular Dynamic (VMD) software version 1.9 [40] and UCSF Chimera [41] were used to visualize the simulation results. All of the molecular graphical presentations were generated using the UCSF Chimera package.

**Table 1:** General description of prepared 13 systems for MD simulation. Simulations are including Aβ17–42, and Aβ17–42 with diferent number of flavonoid molecules including 1, 2, 6 and 10. Subscripts denote the molar ratios for flavonoids/Aβ17–42. Aβ-Myc: Aβ-Myricetin, Aβ-Mor: Aβ-Morin, Aβ-Flv: Aβ-Flavone.

Systems	System size (atom)	Water molecules
Aβ	17214	5656
Aβ-Myc_1_	17052	5591
Aβ-Myc_2_	17046	5578
Aβ-Myc_6_	20296	6616
Aβ-Myc_10_	20407	6609
Aβ-Mor_1_	17054	5592
Aβ-Mor_2_	17044	5578
Aβ-Mor_6_	18227	5929
Aβ-Mor_10_	18358	5930
Aβ- Flv_1_	17069	5599
Aβ- Flv_2_	17065	5589
Aβ- Flv_6_	17043	5547
Aβ- Flv_10_	17171	5555

### MM-PBSA binding free energy

Gibbs free energies of flavonoids/Aβ17–42 complexes were calculated using MM-PBSA (molecular mechanics Poisson Boltzmann surface area) tool of Gromacs method [42]. The binding affinities were investigated during equilibrium phase by taking 200 snapshots at an interval of 100 ps from 80 to 100 ns MD simulations. Therefore, for each simulated system, total 200 snapshots were taken from the last 20 ns of the trajectory. This method is widely used for estimating binding free energies of ligand-protein complex in solvent from the snapshots of MD trajectory based on the estimation by;

$${\Delta G}_{binding}=G_{complex}-{(G}_{A\beta 17-42}+G_{flavonoid})$$

Where G_complex_ is the total free energy of the Aβ-flavonoid complex, G_Aβ17–42_ and G_flavonoid_ are total energy of separated Aβ17–42 and flavonoid in solvent, respectively.

## Results and discussion

### Assessment of conformational stability of Aβ17–42

**Fig 3:**
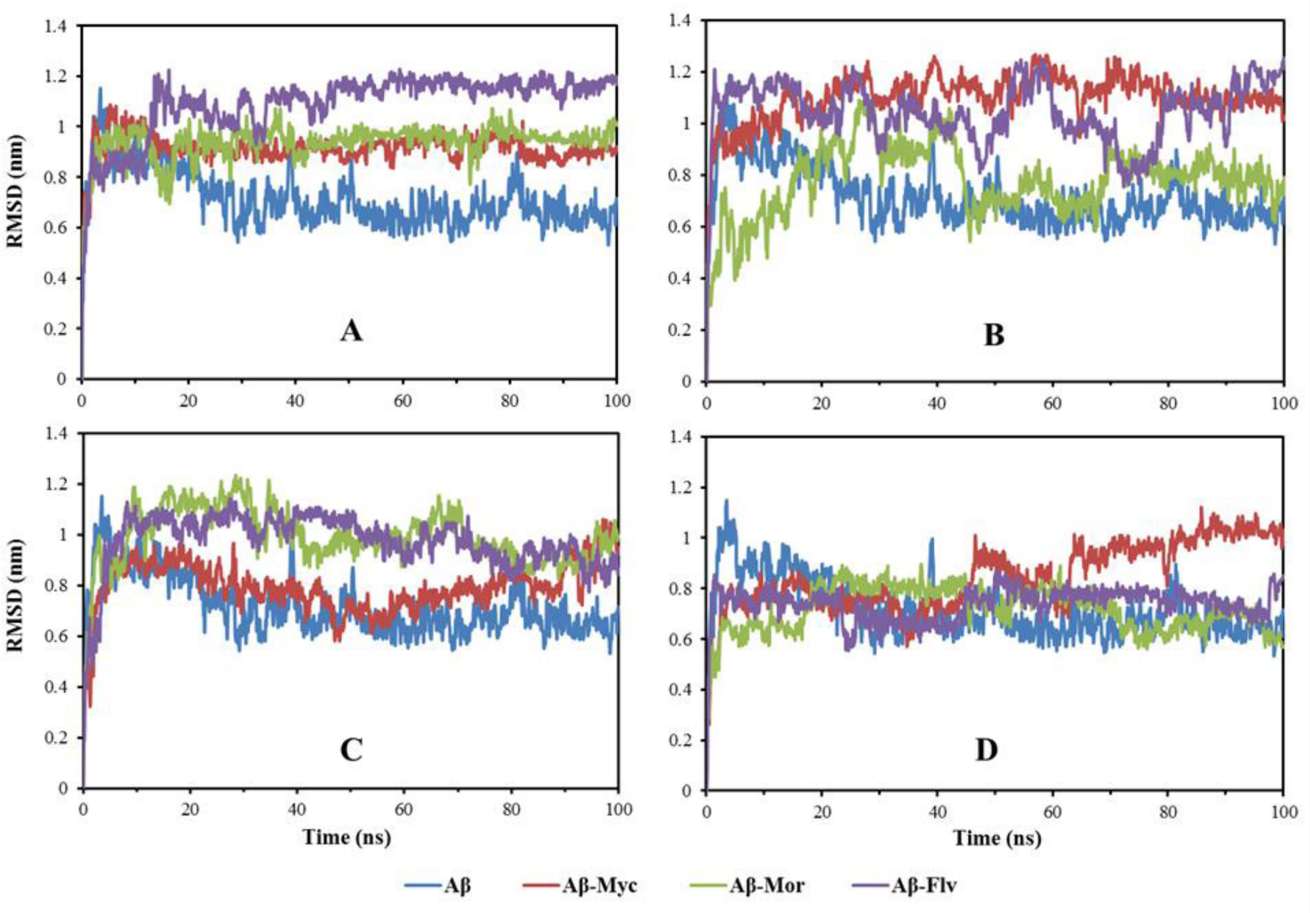
Variation of backbone root mean square deviation (RMSD). **A-D** denote that the molar ratios for flavonoids/Aβ42 are 1, 2, 6 and 10, respectively (**A**; 1-ligand treated system, **B**; 2-ligands treated system, **C**; 6-ligands treated system and **D**; 10-ligands treated system. Aβ-Myc: Aβ-Myricetin, Aβ-Mor: Aβ-Morin, Aβ-Flv: Aβ-Flavone.

**Fig 4:**
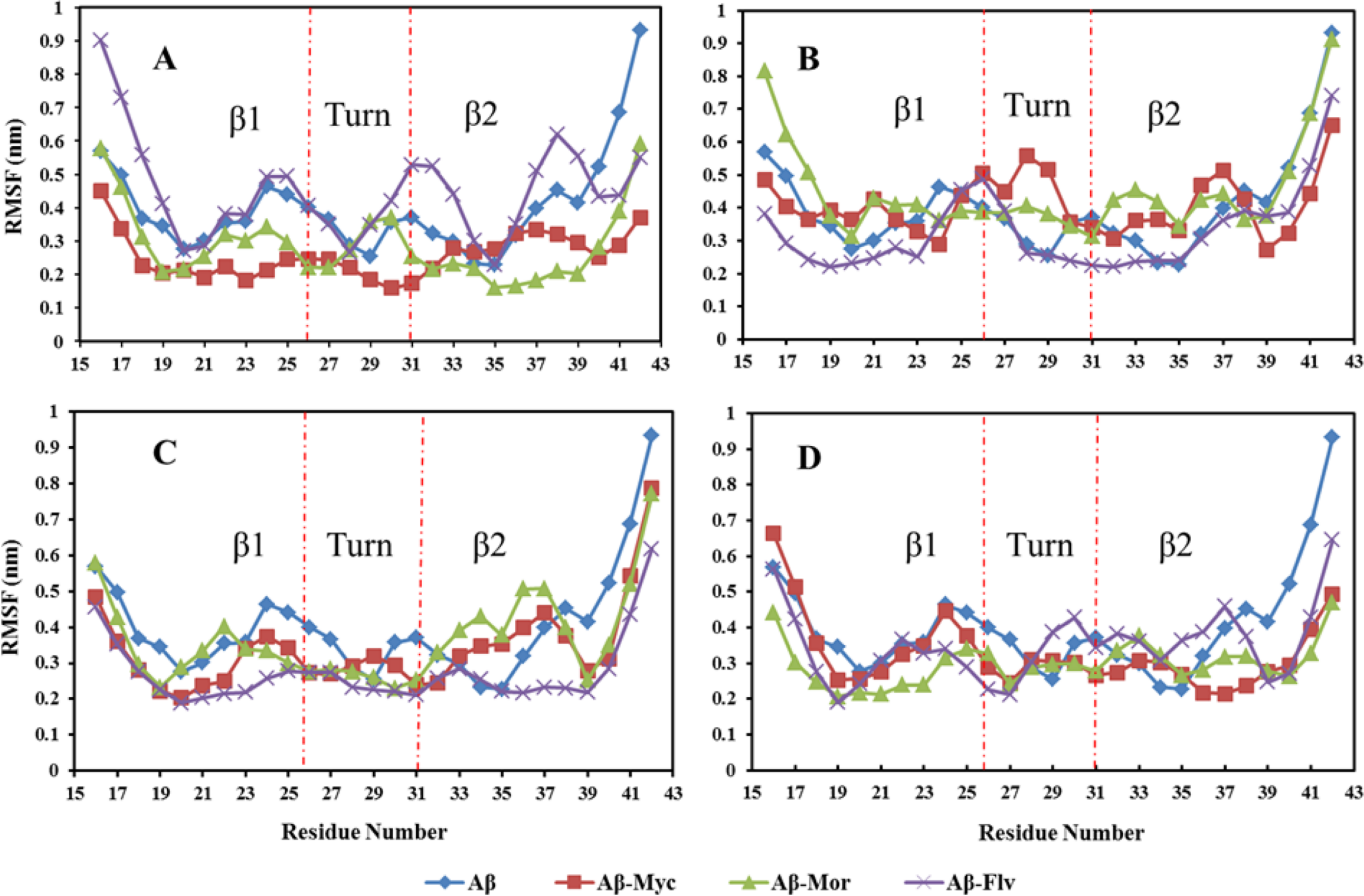
Root mean square fluctuation (RMSF) of Aβ17–42 in different segments (β1, Turn, β2) as a function of time. N-terminal β-strand (termed β1, residues 18–26), C-terminal β-strand (β2, residues 31–42), with a turn region connecting the two strands. A-D denote that the molar ratios for flavonoids/Aβ17–42 are 1, 2, 6 and 10, respectively.

The conformational features of free and complex Aβ17–42 were evaluated by the root-mean squared deviation (RMSD) and root mean square fluctuation (RMSF) assays. The backbone RMSD of Aβ17–42 in different systems has been shown in Fig 3. It shows that the systems have been already equilibrated during simulations and would be possible for additional analyzing at the atomic scale. Almost all complex systems have larger structural changes compared to free system of Aβ17–42. The root mean square fluctuation (RMSF) values over the time course of MD simulation, indicate the intricate behavior of different segments of Aβ17–42 peptide in the presence of flavonoids (Fig 4). Different flavonoids in various mole ratios change the backbone flexibility of Aβ17–42 peptide. The applied flavonoids resulted in increasing backbone rigidity of some residues and also induced more fluctuations for other groups. It means that the interaction of ligands’ functional groups with some residues causes an increase in the rigidity of peptide’s backbone and fluctuation of non-interacted residues. Moreover, different mole ratios of ligands change the platform of flexibility plane of Aβ17–42 peptide in β1, turn and β2 regions. At high mole ratios of ligands, the flexibility behavior of peptide in different regions is almost in homogenous manner. Applying 6:1 and mainly 10:1 mole ratios of different ligands (myricetin, morin and flavone) to Aβ17–42 resulted in almost similar backbone flexibility pattern (Fig 4). RMSF deviations of Aβ-myricetin, Aβ-morin and Aβ-flavone systems are the smallest at 10:1 mole ratio. Therefore, it is expected that the destabilizing Aβ structure and toxicity reduction effectiveness by different natural flavonoids will be associated with similar functionality at higher molar ratios. In other words, different flavonoid compounds have similar conformational feature change effects at high molar ratios.

### H-bond formation possibility between Aβ17–42 and flavonoids

**Fig 5:**
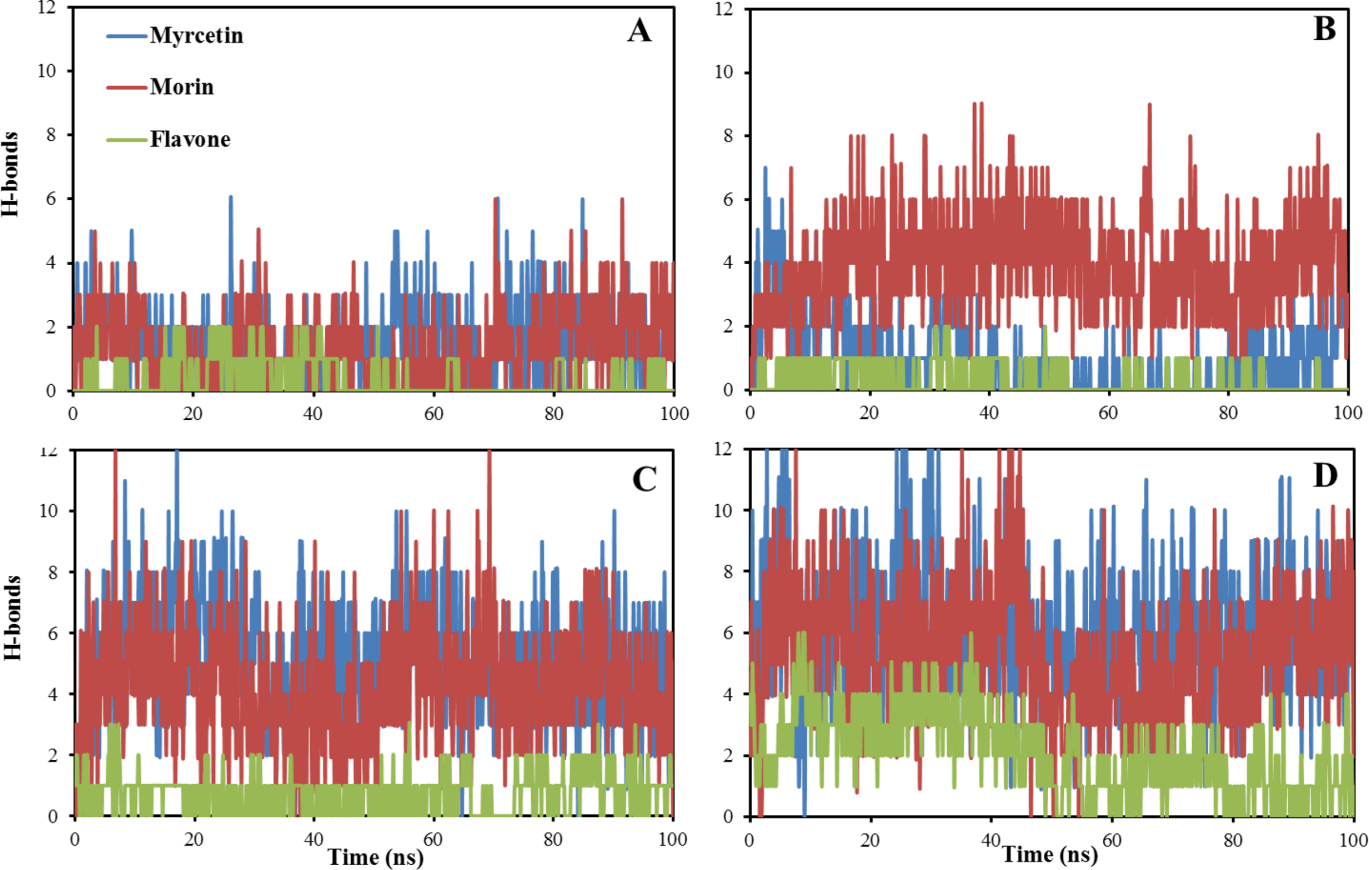
The number of H-bonds between the different mole ratios of flavonoids and Aβ as a simulation time evolution. The distance between H-bond donor and acceptor atoms for H-bond formation possibility was assigned 0.35nm [53]. A-D denote that the molar ratios for flavonoids/Aβ42 are 1, 2, 6 and 10, respectively.

**Fig 6:**
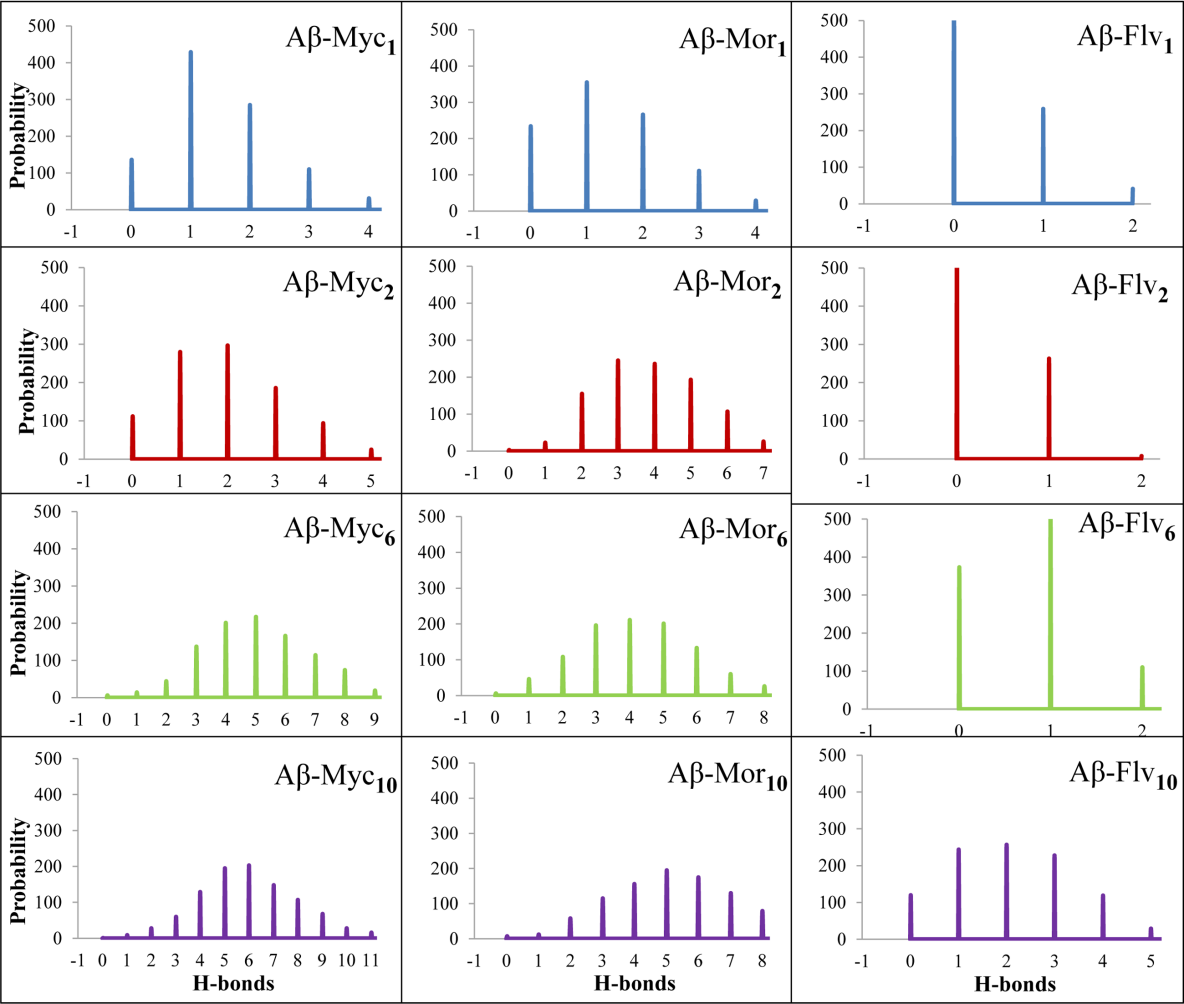
Histograms the number of intermolecular H-bonds vs the probability of occurrence in different systems between flavonoids-Aβ42. Subscripts refer to the corresponding flavonoids numbers.

Experimental and theoretical studies have suggested that the strong affinity between protein/polyphenols is driven by hydrogen-bonding interactions and possibly hydrophobic contacts [43]. Fig 5 clearly displays the variation of hydrogen bonds between different mole ratios of flavonoids and the peptide backbone. It shows all of flavonoids have the ability to form H-bonds with Aβ17–42 peptide backbone during the simulations. Increasing the mole ratios of flavonoids caused an increase in the formation of H-bonds. Therefore, decreasing the backbone flexibility of Aβ17–42 peptide by applying different flavonoids in Fig 4 should be attributed to the intermolecular hydrogen bond formation of ligands with Aβ throughout the 100 ns trajectories. In addition, the formation of hydrogen bonds between flavonoids and the peptide backbone, indicates the competition of flavonoids for backbone H-bonds. Myricetin and morin (with 6 and 5 OHs, respectively) have high possibility of intermolecular H-bonds formation compared to flavone (with no OH). However, flavone can participate in intermolecular H-bonds with Aβ17–42 peptide backbone because of having H-bond acceptors of carbonyl (C = O) and ether (R-O-R) groups. These groups of flavone molecule will be attracted to partial positive charge of H atom in peptide amine (NH_2_) since the nitrogen of peptide amine is highly negative. Computations of the electronic structure of amides and the peptide bond showed that the partial negative charge values of the nitrogen atom in peptide bonds are varying between the range of -0.20 to -1.23 depending on the applied force field and/or basis set [44]. The partial negative charge on nitrogen atom is because of the relatively electron negative nitrogen atom attracting electrons toward itself from adjacent atoms. Therefore, carbonyl and ether groups of flavone act as H-bond acceptors, accepting H atom from NH_2_ group of Aβ17–42 peptide backbone. But, the hydroxyl groups of myricetin and morin have the original possibility to interact with the peptide’s carbonyl (C = O) and amine (NH_2_) groups serving as the hydrogen bond donors. Consequently, data clearly denote that the flavonoids with hydroxyl group (myricetin and morin) makes H-bonds with both peptide’s carbonyl and amine groups but flavone with no hydroxyl substitution group forms H-bonds only with peptide’s amine portion. Formation of intermolecular H-bonds means the disruption of peptide structure by losing peptide’s intramolecular backbone H-bonds between carbonyl and amine groups as important parts for the helical/sheet structure content and stability of proteins. Therefore, using polyphenols such as myricetin or morin would be more effective in destabilizing β-sheet structures of peptide compared to flavone. Analysis of the histogram plots of H-bonds in Fig 6 indicates that the normal distribution of H-bonds is corresponding to mole ratios of flavonoids. Increasing the number of flavonoids resulted in better distribution and their normality seems good. At low mole ratios of ligands/Aβ17–42 (1:1 and almost 2:1), the H-bonds histograms of all myricetin, morin and flavone have non-normal distributions. Polyphenolic myricetin and morin show better normality of H-bonds distribution compared to flavone. At the mole ratios of 6 and 10, both myricetin and morin show normal H-bonds distributions. Nevertheless, flavone shows normal distribution of H-bond histogram only at the mole ratio of 10. It should be noted that, the normal distribution is very important in probability theory [45]. Therefore, based on the probability theory, flavonoids with more OHs are better candidate to interact and disrupt Aβ structure because of providing Gaussian distribution at the lower concentrations.

### Free energies of flavonoids/Aβ17–42 complexes

**Fig 7:**
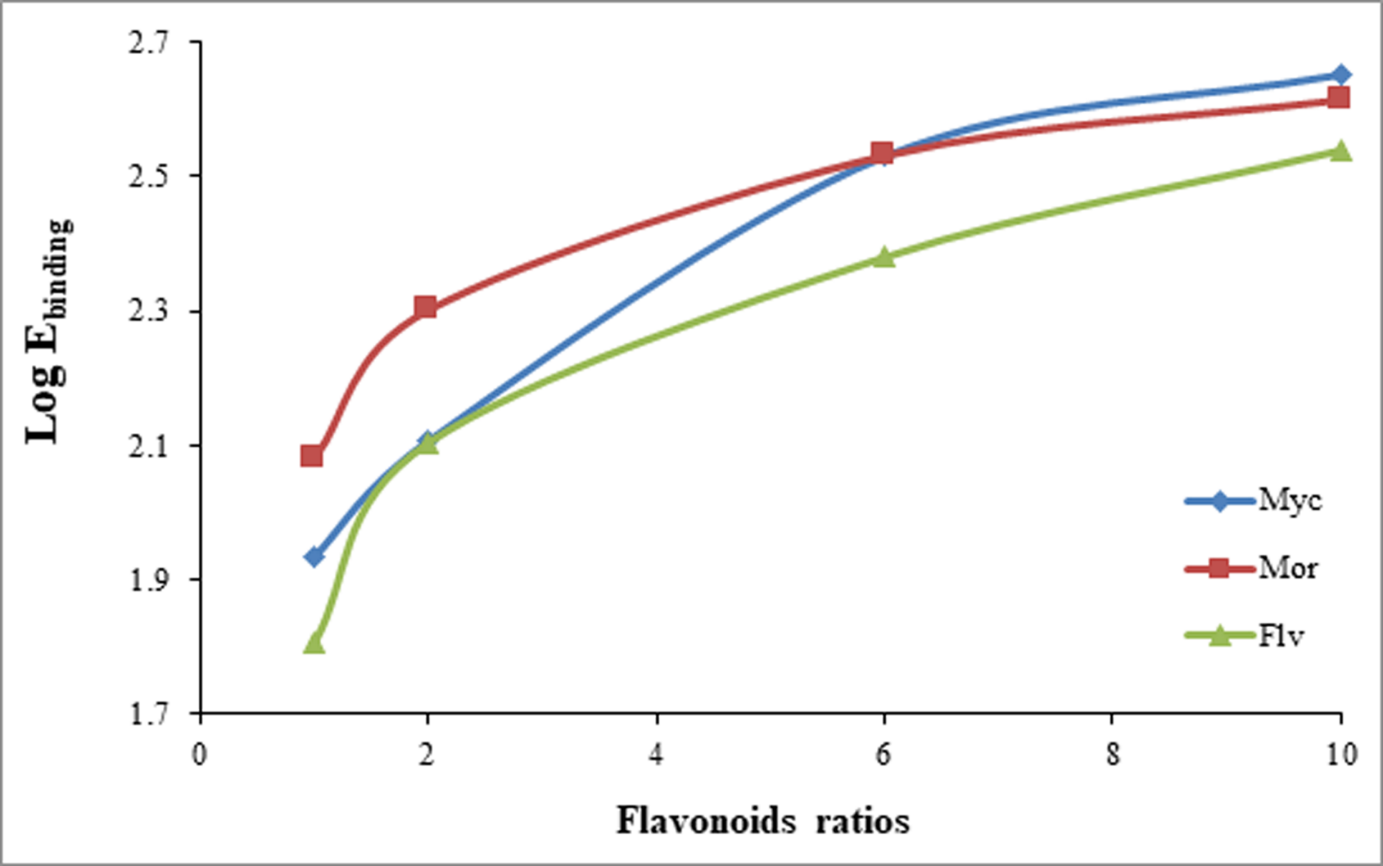
Semi-log plots of binding energy (kJ/mol) vs ligands moles achievd by MM/PBSA method. The absolute values of binding energy applied for Log E_binding_.

The binding energies obtained from the MM/PBSA calculation of different flavonoids/Aβ17–42 complexes are listed in Table 2. The favorable interaction energies including van der Waals, electrostatic and non-polar solvation energy (solvent accessible surface area, SASA energy) negatively contribute to the total binding free energies. These energies altogether contribute to the flavonoids/Aβ17–42 complex stability. The results indicate that polyphenolic myricetin and morin possessed more negative binding free energies than flavone for all of the applied mole ratios. In all cases, the contribution of van der Waals interactions in the total interaction energy is much larger than the electrostatic energies. Also, non-polar SASA energies contribute less than electrostatic energies in the total binding energies of all systems. The transfer energies of compounds from a nonpolar solvent to water are described by SASA-dependent energy function [42]. Then, the hydrophobic nonpolar hydration free energy, ΔG_np_, is decomposed as;

$${\Delta G}_{np}={\Delta G}_{SASA}+{\Delta G}_{vdw}$$

$${\Delta G}_{SASA}=G_{SASA-complex}-{(G}_{SASA-ligand}+G_{SASA-A\beta })$$

where ΔG_SASA_ is the cavity hydration free energy, and ΔG_vdw_ is the free energy for establishing the solute-solvent van der Waals dispersion interactions. However, the cavity formation in water has been frequently used as a model for studying the hydrophobic effect [46]. Hence, non-polar solvation energy is the SASA-dependent hydrophobic solvation energy. While the solute-solvent van der Waals interactions are dominant in Table 2, the pure hydrophobic interactions related to SASA-dependent energy are trivial in all complexes. Moreover, logP values (lipophilicity parameter) of different species refer to larger hydrophobic property of flavone (between 2 and 2.5-fold) compared to polyphenolic flavonoids of myricetin or morin (logP_flavone_ 3.56; logP_myricetin_ 1.42; logP_morin_ 1.54; https://www.ncbi.nlm.nih.gov/pccompound). Although flavone is a more hydrophobic compound, its binding energy (Table 2) is certainly less than myricetin or morin compounds. It means that the hydrophobic interactions of flavonoids with Aβ17–42 should be negligible. Moreover, binding energy plots of all systems (Fig 7) clearly indicate the hyperbolic behavior of flavonoids binding. Since non-cooperative interaction is hyperbolic and the cooperative hydrophobic response is sigmoidal shape [47, 48], then the nonsigmoid-type of interactions in all applied flavonoids denote the insignificant role of hydrophobic interactions for myricetin, morin and flavone with Aβ17–42. Consequently, we believe the role of H-bonds is dominant as clearly discussed in previous section.

**Table 2:** Summary of calculated molecular interactions of the flavonoids with Aβ17–42 using MM/PBSA tools. Subscripts denote the molar ratios for flavonoids/Aβ17–42. Aβ-Myc: Aβ-Myricetin, Aβ-Mor: Aβ-Morin, Aβ-Flv: Aβ-Flavone.

Systems	Van der waal(kJ/mol)	Electrostattic (kJ/mol)	Polar salvation (kJ/mol)	SASA energy (kJ/mol)	Binding energy (kJ/mol)
**Aβ-Myc_1_**	-129.15 +/-73.23	-14.94 +/-11.74	70.65 +/-42.40	-11.81 +/-6.57	-85.25 +/- 49.58
**Aβ-Mor_1_**	-158.81 +/-8.96	-31.185 +/-10.25	83.76 +/-13.57	-13.24 +/-0.71	-119.47 +/11.02
**Aβ- Flv_1_**	-99.01 +/-13.25	-21.17 +/-13.58	65.78 +/-23.71	-9.82 +/-1.26	-64.23 +/- 18.65
**Aβ-Myc_2_**	-160.37 +/-29.39	-24.38 +/-14.66	74.85 +/-26.79	-17.27 +/-2.66	-127.16 +/- 24.42
**Aβ-Mor_2_**	-264.80 +/-28.96	-42.33 +/-11.05	131.39 +/-20.17	-23.86 +/-1.71	-199.61 +/- 27.54
**Aβ- Flv_2_**	-148.73 +/-31.18	-7.10 +/-10.12	42.85 +/-23.24	-13.94 +/-2.46	-126.92 +/- 27.00
**Aβ-Myc_6_**	-464.59 +/-32.09	-55.91 +/-25.40	221.62 +/-36.80	-41.08 +/-2.76	-339.97 +/- 28.54
**Aβ-Mor_6_**	-448.75 +/-27.44	-65.49 +/-18.76	222.39 +/-28.02	-41.42 +/-2.28	-333.28 +/- 26.80
**Aβ- Flv_6_**	-321.84 +/-28.06	-39.71 +/-17.72	149.39 +/- 35.50	-27.74 +/-2.26	-239.94 +/- 27.01
**Aβ-Myc_10_**	-627.52 +/-24.24	-121.09 +/-20.77	352.27 +/-30.29	-54.00 +/-2.00	-450.37 +/- 28.27
**Aβ-Mor_10_**	-535.08 +/-27.92	-88.00 +/-23.31	258.49 +/-45.34	-47.07 +/-2.13	-411.69 +/- 32.55
**Aβ- Flv_10_**	-485.34 +/-42.70	-35.14 +/-27.47	215.01 +/-45.35	-40.76 +/-3.11	-346.17 +/- 50.50

### Secondary structure analysis

**Fig 8:**
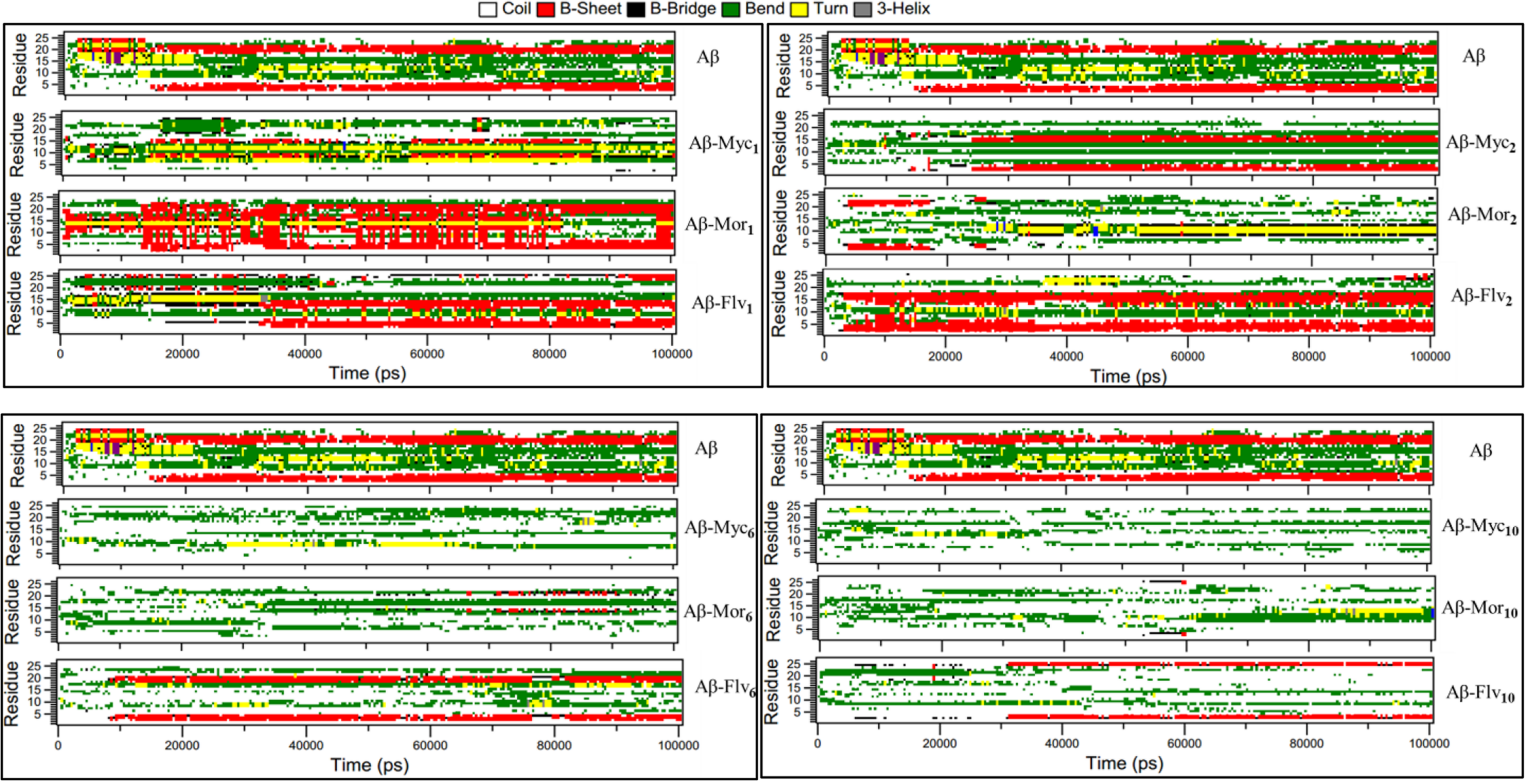
Computing the secondary structure from PDB entries for each time frame calling the dssp program. Secondary structural changes using dssp during molecular dynamics simulations of the systems in different mole ratios of falvonids Aβ were achieved. Aβ, Aβ-Myc, Aβ-Mor, and Aβ-Flv correspond to Aβ and Aβ in complex with myricetin, morin and flavonoid, respectively. Subscripts refer to the corresponding flavonoids numbers.

Secondary structure content including alpha helical and β-sheet structures are essential for amyloidosis and neurotoxicity [49, 50]. Any agent with capability to prevent and inhibit conversion of alpha helical to β sheet structure could be a useful candidate for AD treatment [51]. To evaluate any affinity of the applied compounds to disrupt the content of β sheet structure in Aβ17–42 peptide, secondary structure analysis was accomplished by DSSP [52]. Fig 8 clarifies the temporal expansion of the secondary structure content for different systems along 100 ns trajectory. The results of secondary structure contents of systems entangled with different mole ratios of morin are almost similar to myricetin systems. The morin and myricetin treated systems exhibit that the β-sheet conformations have been disappeared at higher mole ratios. In flavone treated systems, the contents of β-sheet structures between β1 and β2 regions are reduced by increasing flavone’s mole ratios. Data certainly indicate that losing the β-sheet contents of Aβ corresponds to the ligands mole ratios mainly the normality of intermolecular H-bonds distribution. Systems with normal distribution of intermolecular H-bonds clearly correspond to the suppression of any appearance of β-sheet structures. As discussed and examined at Fig 6, two mole ratios of myricetin and morin (6 and 10) and one mole ratio of flavone (10) denote normality manner in intermolecular H-bonding, namely, normal systems. The normal systems are active against β-sheet structures, resulted in losing beta sheet conformations. All of treated systems with different flavonoids exhibit other types of secondary structures mainly coil and bend conformations. S1 Table clearly shows that the initial β-sheet, β-bridge, bend and turn contents in untreated system are exchanged for random coil elements in complex systems. In this process morin content system is mainly accompanying conversion of β-sheet structure into bend but myricetin resulted in β-sheet conversion into coil conformations. The complete loss of beta structures mainly in morin and myricetin treated systems are more noticeable, indicating that, polyphenolic flavonoids such as morin and myricetin could have the greatest impact on secondary structure content and suppress β-sheet formation toward β-amyloids.

### Tertiary structure and density profile of the systems

**Fig 9:**
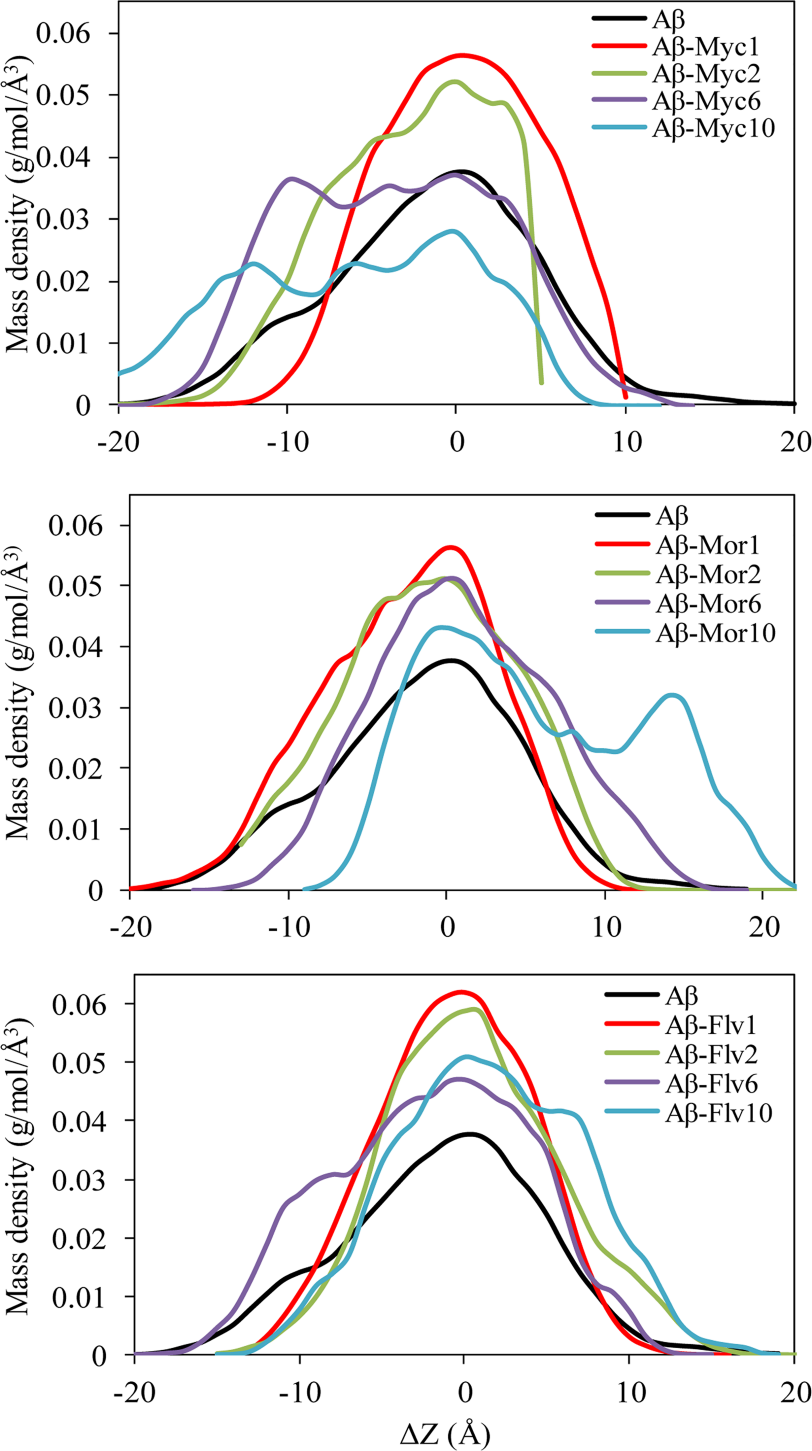
1-D projections of mass density profile along the z axis (resolution = 1 Å) for the Aβ peptide in free ligands system and the complexs with 1, 2, 6 and 10 ligands. The amount of |Δz| corresponds to the portions of thickness from dense core at Δz≈0. The distance from Δz = 0 is a direct indicator protein widths or unfolded state. Subscripts refer to the corresponding flavonoids numbers. All computations correspond to the last 20 ns of each trajectory.

**Fig 10:**
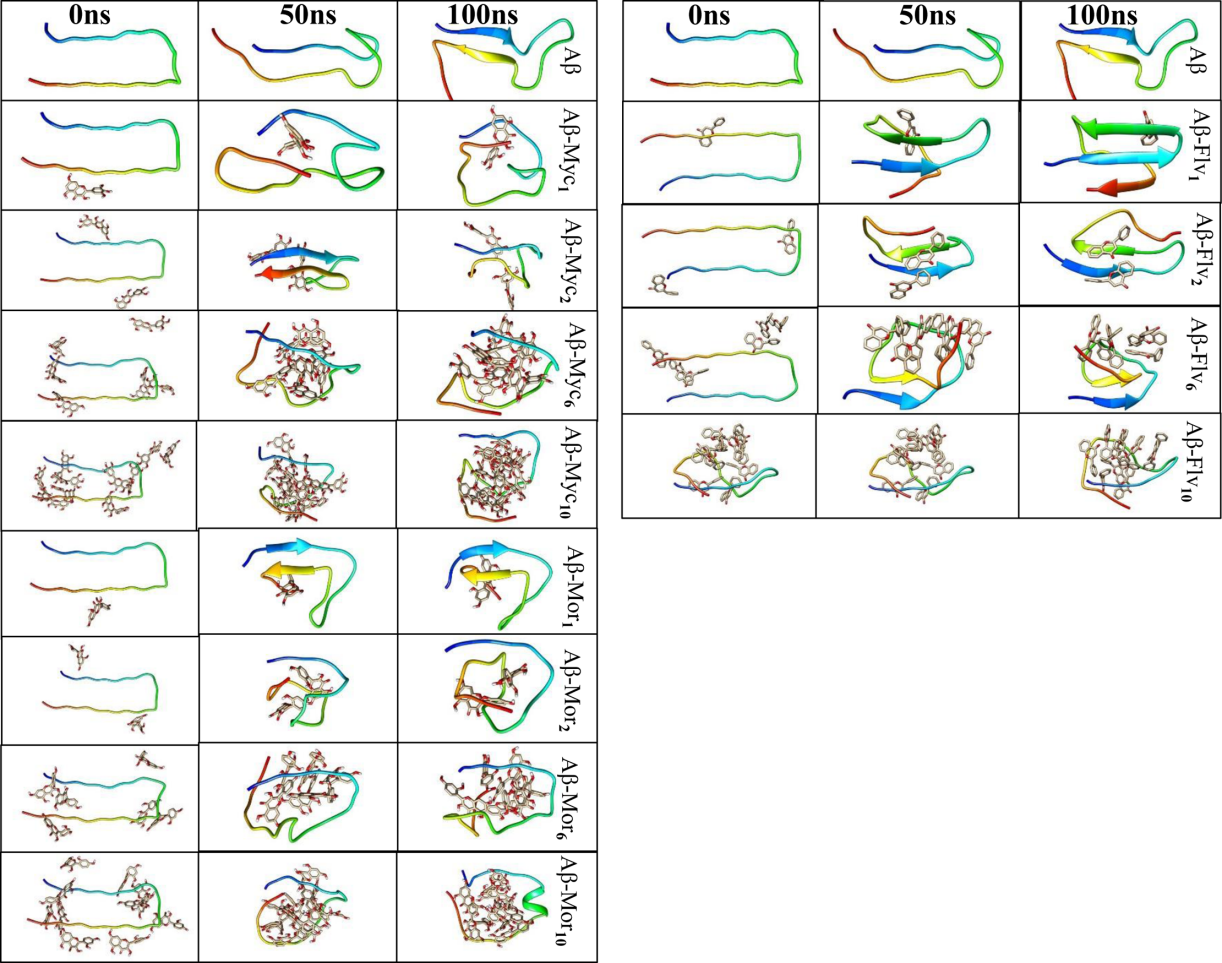
Snapshots of Aβ in absence and presence of different mole ratios of myricetin (Myc), morin (Mor), and flavone (Flv) at the beginning, 50 and 100ns of MD simulation. Subscripts refer to the corresponding flavonoids numbers. The graphical presentations were created by UCSF Chimera.

Density function, such as mass or number densities, an analysis tool that can be derived from MD trajectories to compute one-dimensional density profiles of molecular systems. In order to evaluate any possible changes in tertiary structure of Aβ peptide by entangling various mole ratios of flavonoids, 1-D projections of peptide mass density profile along the z axis was analyzed and monitored at Fig 9. The flavonoid free system has normal distribution profile meaning that all parts of Aβ peptide structures are benefited from similar structure density at the definite range of Δz. Applying “types of flavonoids” and their “mole ratios” resulted in changing the peptide mass density profile. Myricetin and morin at the ratios of 6 and 10 caused Aβ peptide completely to unfold with increasing the peptides portions of thickness from dense core (increasing |Δz| and appearing new peaks). The process is concomitant to loss of the normal distribution of peptide mass density profile. It means that myricetin and morin caused 3D structure of Aβ peptide completely to change at the molar ratios of 6 and 10. Almost similar results were achieved for flavone at the molar ratio of 10. While, all of the treated systems with one and two flavonoids of myricetin, morin and flavone showed higher density profile. Increasing peptide mass density at low mole ratios of ligands did not demonstrate any noticeable new peak. The rigidity of Aβ peptide was obviously increased in complexed systems containing one and two ligands at all segments of the structure. Different snapshots of free Aβ and in complex with different mole ratios of myricetin, morin and flavone were prepared and shown at Fig 10 for the MD simulations. Although all systems were initially prepared by placing ligands on U-shaped conformation surface of Aβ peptide (surrounding ~4 Å), the ligands were shifted inside of the peptide throughout simulations. This is mainly because of ligands tendency to interact with Aβ peptide and mainly to participate intermolecular backbone H-bonds formation with negative binding energies. The non-normal distribution of H-bonds at low mole ratios of flavonoids (Fig 6) is accompanying to firm trivial segments of Aβ conformation. Therefore, the systems containing one and two flavonoids resulting mass densities increase at small range of ΔZ. But, the systems with normal H-bonds distribution at high mole ratios of flavonoids correspond to involve almost all segments of peptides by H-bond formation capability interactions at a wider range of ΔZ (Fig 6 and Fig 9). In addition, the geometric form of Aβ peptide in different snapshots clearly support the density profile and H-bond histogram data. It shows that at higher mole ratios of flavonoids the conformation of Aβ peptide completely changed toward embracing flavonoids. Flavonoids penetrate the core of the Aβ17–42, forming large self-aggregated clusters. Because of the formation of Aβ17-42-ligands clusters, the peptide mass density profile was changed and caused to lose the sharpness and maximum values of mass density concerning wide range of ΔZ.

Finally, the important findings of this manuscript are concluding that; a) all of applied flavonoids including myricetin, morin polyphenolic and flavone are mainly capable of binding to all regions of Aβ peptide including β1, β2, and turn segments. These interactions are favorable and negatively contribute to the total binding free energies. Binding affinity of flavonoids to different segment of peptide for disrupting Aβ peptide structure is essentially because of the possessing H-bond donor and acceptor groups in flavonoids structure including ether, carbonyl and hydroxyl. b) polyphenolic flavonoids with more functional OHs (myricetin and morin) are more active against β-sheet structures destabilization capability. c) The main effects of flavonoids against beta amyloid or Aβ aggregation correspond to the good distribution of H-bonds in the complex systems. The good distribution of H-bonds corresponds to both of the flavonoids’ mole ratios and the number of their functional OH groups. Hence, flavonoids with more functional OH groups and Gaussian distribution at low mole ratio are better applicable to interact and disrupt Aβ structure. Since polyphenolic flavonoids are beneficial in inhibiting β-sheet and preventing the elongation of Aβ oligomers, the compounds myricetin and morin can be introduced as a useful candidate for AD treatment.

## Supporting information

S1 Table Percentage of the secondary structural contents of Aβ-monomer in each system during 100ns simulation. (DOCX)

## References

[1] Cohen FE, Kelly JW. Therapeutic approaches to protein-misfolding diseases. Nature. 2003;426(6968):905–9. doi: 10.1038/nature02265 14685252 10.1038/nature02265

[2] Hardy JA, Higgins GA. Alzheimer's disease: the amyloid cascade hypothesis. Science. 1992;256(5054):184 1566067 10.1126/science.1566067

[3] Dumoulin M, Johnson RJ, Bellotti V, Dobson CM. Human lysozyme. Protein Misfolding, Aggregation, and Conformational Diseases: Springer; 2007 p. 285–308.

[4] Chiti F, Dobson CM. Protein misfolding, functional amyloid, and human disease. Annu Rev Biochem. 2006;75:333–66. doi: 10.1146/annurev.biochem.75.101304.123901 16756495 10.1146/annurev.biochem.75.101304.123901

[5] Ross CA, Poirier MA. Protein aggregation and neurodegenerative disease. 2004. 10.1038/nm1066 15272267

[6] Hardy J, Allsop D. Amyloid deposition as the central event in the aetiology of Alzheimer's disease. Trends in pharmacological sciences. 1991;12:383–8. 1763432 10.1016/0165-6147(91)90609-v

[7] Brookmeyer R, Johnson E, Ziegler-Graham K, Arrighi HM. Forecasting the global burden of Alzheimer’s disease. Alzheimer's & dementia. 2007;3(3):186–91. 10.1016/j.jalz.2007.04.381 19595937

[8] Harrington CR. The molecular pathology of Alzheimer's disease. Neuroimaging Clinics of North America. 2012;22(1):11–22. doi: 10.1016/j.nic.2011.11.003 22284730 10.1016/j.nic.2011.11.003

[9] Haass C, Selkoe DJ. Soluble protein oligomers in neurodegeneration: lessons from the Alzheimer's amyloid β-peptide. Nature reviews Molecular cell biology. 2007;8(2):101–12. doi: 10.1038/nrm2101 17245412 10.1038/nrm2101

[10] Cheon M, Kang M, Chang I. Polymorphism of fibrillar structures depending on the size of assembled Aβ 17–42 peptides. Scientific reports. 2016;6:38196 doi: 10.1038/srep38196 27901087 10.1038/srep38196 PMC5128875

[11] Cheon M, Hall CK, Chang I. Structural conversion of Aβ17–42 peptides from disordered oligomers to u-shape protofilaments via multiple kinetic pathways. PLoS computational biology. 2015;11(5):e1004258 doi: 10.1371/journal.pcbi.1004258 25955249 10.1371/journal.pcbi.1004258 PMC4425657

[12] Saura CA, Choi S-Y, Beglopoulos V, Malkani S, Zhang D, Rao BS, et al Loss of presenilin function causes impairments of memory and synaptic plasticity followed by age-dependent neurodegeneration. Neuron. 2004;42(1):23–36. 15066262 10.1016/s0896-6273(04)00182-5

[13] Barten DM, Albright CF. Therapeutic strategies for Alzheimer’s disease. Molecular neurobiology. 2008;37(2–3):171–86. doi: 10.1007/s12035-008-8031-2 18581273 10.1007/s12035-008-8031-2

[14] Ono K, Hamaguchi T, Naiki H, Yamada M. Anti-amyloidogenic effects of antioxidants: implications for the prevention and therapeutics of Alzheimer's disease. Biochimica et Biophysica Acta (BBA)-Molecular Basis of Disease. 2006;1762(6):575–86. 16644188 10.1016/j.bbadis.2006.03.002

[15] Lemkul JA, Bevan DR. Morin inhibits the early stages of amyloid β-peptide aggregation by altering tertiary and quaternary interactions to produce “off-pathway” structures. Biochemistry. 2012;51(30):5990–6009. doi: 10.1021/bi300113x 22762350 10.1021/bi300113x

[16] Villmow M, Baumann M, Malesevic M, Sachs R, Hause G, Fändrich M, et al Inhibition of Aβ (1–40) fibril formation by cyclophilins. Biochemical Journal. 2016;473(10):1355–68. doi: 10.1042/BCJ20160098 26994210 10.1042/BCJ20160098

[17] Lemkul JA, Bevan DR. Destabilizing Alzheimer’s Aβ42 protofibrils with morin: mechanistic insights from molecular dynamics simulations. Biochemistry. 2010;49(18):3935–46. doi: 10.1021/bi1000855 20369844 10.1021/bi1000855

[18] Ono K, Yoshiike Y, Takashima A, Hasegawa K, Naiki H, Yamada M. Potent anti‐amyloidogenic and fibril‐destabilizing effects of polyphenols in vitro: implications for the prevention and therapeutics of Alzheimer's disease. Journal of neurochemistry. 2003;87(1):172–81. 12969264 10.1046/j.1471-4159.2003.01976.x

[19] Fitzpatrick AW, Debelouchina GT, Bayro MJ, Clare DK, Caporini MA, Bajaj VS, et al Atomic structure and hierarchical assembly of a cross-β amyloid fibril. Proceedings of the National Academy of Sciences. 2013;110(14):5468–73. 10.1073/pnas.1219476110 PMC3619355 23513222

[20] Dong M, Paul TJ, Hoffmann Z, Chan K, Hu D, Ai H, et al Structural and Material Properties of Amyloid Aβ40/42 Fibrils. ChemPhysChem. 2016;17(16):2558–66. doi: 10.1002/cphc.201600256 27146076 10.1002/cphc.201600256

[21] Fan H-M, Gu R-X, Wang Y-J, Pi Y-L, Zhang Y-H, Xu Q, et al Destabilization of Alzheimer’s Aβ42 protofibrils with a novel drug candidate wgx-50 by molecular dynamics simulations. The Journal of Physical Chemistry B. 2015;119(34):11196–202. doi: 10.1021/acs.jpcb.5b03116 25996452 10.1021/acs.jpcb.5b03116

[22] Zheng J, Jang H, Ma B, Tsai C-J, Nussinov R. Modeling the Alzheimer Aβ17–42 fibril architecture: tight intermolecular sheet-sheet association and intramolecular hydrated cavities. Biophysical journal. 2007;93(9):3046–57. doi: 10.1529/biophysj.107.110700 17675353 10.1529/biophysj.107.110700 PMC2025669

[23] Kumar A, Srivastava S, Tripathi S, Singh SK, Srikrishna S, Sharma A. Molecular insight into amyloid oligomer destabilizing mechanism of flavonoid derivative 2-(4′ benzyloxyphenyl)-3-hydroxy-chromen-4-one through docking and molecular dynamics simulations. Journal of Biomolecular Structure and Dynamics. 2016;34(6):1252–63. doi: 10.1080/07391102.2015.1074943 26208790 10.1080/07391102.2015.1074943

[24] Akaishi T, Morimoto T, Shibao M, Watanabe S, Sakai-Kato K, Utsunomiya-Tate N, et al Structural requirements for the flavonoid fisetin in inhibiting fibril formation of amyloid β protein. Neuroscience letters. 2008;444(3):280–5. doi: 10.1016/j.neulet.2008.08.052 18761054 10.1016/j.neulet.2008.08.052

[25] Zhu JT, Choi RC, Chu GK, Cheung AW, Gao QT, Li J, et al Flavonoids possess neuroprotective effects on cultured pheochromocytoma PC12 cells: a comparison of different flavonoids in activating estrogenic effect and in preventing β-amyloid-induced cell death. Journal of Agricultural and Food Chemistry. 2007;55(6):2438–45. doi: 10.1021/jf063299z 17323972 10.1021/jf063299z

[26] Ho L, Chen LH, Wang J, Zhao W, Talcott ST, Ono K, et al Heterogeneity in red wine polyphenolic contents differentially influences Alzheimer's disease-type neuropathology and cognitive deterioration. Journal of Alzheimer's Disease. 2009;16(1):59–72. doi: 10.3233/JAD-2009-0916 19158422 10.3233/JAD-2009-0916 PMC2857553

[27] Wang J, Ho L, Zhao W, Ono K, Rosensweig C, Chen L, et al Grape-derived polyphenolics prevent Aβ oligomerization and attenuate cognitive deterioration in a mouse model of Alzheimer's disease. The Journal of Neuroscience. 2008;28(25):6388–92. doi: 10.1523/JNEUROSCI.0364-08.2008 18562609 10.1523/JNEUROSCI.0364-08.2008 PMC2806059

[28] Berhanu WM, Masunov AE. Atomistic mechanism of polyphenol amyloid aggregation inhibitors: molecular dynamics study of Curcumin, Exifone, and Myricetin interaction with the segment of tau peptide oligomer. Journal of Biomolecular Structure and Dynamics. 2015;33(7):1399–411. doi: 10.1080/07391102.2014.951689 25093402 10.1080/07391102.2014.951689

[29] Khatua P, Sinha SK, Bandyopadhyay S. Size-Dependent Conformational Features of Aβ17–42 Protofilaments from Molecular Simulation Studies. Journal of chemical information and modeling. 2017;57(9):2378–92. doi: 10.1021/acs.jcim.7b00407 28853902 10.1021/acs.jcim.7b00407

[30] Szczepanik AM, Rampe D, Ringheim GE. Amyloid‐β peptide fragments p3 and p4 induce pro‐inflammatory cytokine and chemokine production in vitro and in vivo. Journal of neurochemistry. 2001;77(1):304–17. 11279286 10.1046/j.1471-4159.2001.t01-1-00240.x

[31] Lührs T, Ritter C, Adrian M, Riek-Loher D, Bohrmann B, Döbeli H, et al 3D structure of Alzheimer's amyloid-β (1–42) fibrils. Proceedings of the National Academy of Sciences of the United States of America. 2005;102(48):17342–7. doi: 10.1073/pnas.0506723102 16293696 10.1073/pnas.0506723102 PMC1297669

[32] SchuÈttelkopf AW, Van Aalten DM. PRODRG: a tool for high-throughput crystallography of protein–ligand complexes. Acta Crystallographica Section D: Biological Crystallography. 2004;60(8):1355–63. 15272157 10.1107/S0907444904011679

[33] Oostenbrink C, Villa A, Mark AE, Van Gunsteren WF. A biomolecular force field based on the free enthalpy of hydration and solvation: the GROMOS force‐field parameter sets 53A5 and 53A6. Journal of computational chemistry. 2004;25(13):1656–76. doi: 10.1002/jcc.20090 15264259 10.1002/jcc.20090

[34] Berendsen HJ, Postma JP, van Gunsteren WF, Hermans J. Interaction models for water in relation to protein hydration Intermolecular forces: Springer; 1981 p. 331–42.

[35] Liu F-F, Dong X-Y, He L, Middelberg AP, Sun Y. Molecular insight into conformational transition of amyloid β-peptide 42 inhibited by (−)-epigallocatechin-3-gallate probed by molecular simulations. The Journal of Physical Chemistry B. 2011;115(41):11879–87. doi: 10.1021/jp202640b 21899367 10.1021/jp202640b

[36] Takeda T, Chang WE, Raman EP, Klimov DK. Binding of nonsteroidal anti‐inflammatory drugs to Aβ fibril. Proteins: Structure, Function, and Bioinformatics. 2010;78(13):2849–60. 10.1002/prot.22804 PMC3058518 20635343

[37] Raman EP, Takeda T, Klimov DK. Molecular dynamics simulations of ibuprofen binding to Aβ peptides. Biophysical journal. 2009;97(7):2070–9. doi: 10.1016/j.bpj.2009.07.032 19804739 10.1016/j.bpj.2009.07.032 PMC2756381

[38] Hess B. P-LINCS: A parallel linear constraint solver for molecular simulation. Journal of Chemical Theory and Computation. 2008;4(1):116–22. doi: 10.1021/ct700200b 26619985 10.1021/ct700200b

[39] Hess B, Bekker H, Berendsen HJ, Fraaije JG. LINCS: a linear constraint solver for molecular simulations. Journal of computational chemistry. 1997;18(12):1463–72.

[40] Humphrey W, Dalke A, Schulten K. VMD: visual molecular dynamics. Journal of molecular graphics. 1996;14(1):33–8. 8744570 10.1016/0263-7855(96)00018-5

[41] Pettersen EF, Goddard TD, Huang CC, Couch GS, Greenblatt DM, Meng EC, et al UCSF Chimera—a visualization system for exploratory research and analysis. Journal of computational chemistry. 2004;25(13):1605–12. doi: 10.1002/jcc.20084 15264254 10.1002/jcc.20084

[42] Kumari R, Kumar R, Consortium OSDD, Lynn A. g_mmpbsa A GROMACS tool for high-throughput MM-PBSA calculations. Journal of chemical information and modeling. 2014;54(7):1951–62. doi: 10.1021/ci500020m 24850022 10.1021/ci500020m

[43] Le Bourvellec C, Renard C. Interactions between polyphenols and macromolecules: quantification methods and mechanisms. Critical reviews in food science and nutrition. 2012;52(3):213–48. doi: 10.1080/10408398.2010.499808 22214442 10.1080/10408398.2010.499808

[44] Milner‐White EJ. The partial charge of the nitrogen atom in peptide bonds. Protein science. 1997;6(11):2477–82. doi: 10.1002/pro.5560061125 9385654 10.1002/pro.5560061125 PMC2143592

[45] Jaynes ET. Probability theory: the logic of science: Cambridge university press; 2003.

[46] Southall NT, Dill KA, Haymet A. A view of the hydrophobic effect. ACS Publications; 2002.

[47] Porter CM, Miller BG. Cooperativity in monomeric enzymes with single ligand-binding sites. Bioorganic chemistry. 2012;43:44–50. doi: 10.1016/j.bioorg.2011.11.001 22137502 10.1016/j.bioorg.2011.11.001 PMC3307832

[48] Denisov IG, Frank DJ, Sligar SG. Cooperative properties of cytochromes P450. Pharmacology & therapeutics. 2009;124(2):151–67. 19555717 10.1016/j.pharmthera.2009.05.011 PMC2753496

[49] Barrow CJ, Zagorski MG. Solution Structures of (Beta) Peptide and Its Constituent Fragments: Relation to Amyloid Deposition. Science. 1991;253(5016):179 1853202 10.1126/science.1853202

[50] Terzi E, Hoelzemann G, Seelig J. Reversible Random Coil-. beta.-Sheet Transition of the Alzheimer. beta.-Amyloid Fragment (25–35). Biochemistry. 1994;33(6):1345–50. 8312252 10.1021/bi00172a009

[51] Salomon AR, Marcinowski KJ, Friedland RP, Zagorski MG. Nicotine inhibits amyloid formation by the β-peptide. Biochemistry. 1996;35(42):13568–78. doi: 10.1021/bi9617264 8885836 10.1021/bi9617264

[52] Kabsch W, Sander C. Dictionary of protein secondary structure: pattern recognition of hydrogen‐bonded and geometrical features. Biopolymers. 1983;22(12):2577–637. doi: 10.1002/bip.360221211 6667333 10.1002/bip.360221211

[53] Beke T, Csizmadia IG, Perczel A. Theoretical study on tertiary structural elements of β-peptides: Nanotubes formed from parallel-sheet-derived assemblies of β-peptides. Journal of the American Chemical Society. 2006;128(15):5158–67. doi: 10.1021/ja0585127 16608352 10.1021/ja0585127

