# Supplementary material for: The role of phenolic OH groups of flavonoid compounds with H-bond formation ability to suppress amyloid mature fibrils by destabilizing β-sheet conformation of monomeric Aβ17-42
Source: PLoS One. 2018 Jun 28;13(6):e0199541. doi: 10.1371/journal.pone.0199541 (PMC6023135; doi:10.1371/journal.pone.0199541)
Supplement: S1 Table — (DOCX) [file pone.0199541.s001.docx]

**S1 Table.**

| Systems | Structure^a^ | Coil | B-Sheet | B-Bridge | Bend | Turn |
| --- | --- | --- | --- | --- | --- | --- |
| Aβ | 31 | 39 | 23 | 1 | 29 | 7 |
| Aβ-Myc_1_ | 25 | 50 | 7 | 4 | 25 | 14 |
| Aβ-Myc_2_ | 17 | 51 | 16 | 1 | 32 | 4 |
| Aβ-Myc_6_ | 3 | 73 | — | — | 23 | 3 |
| Aβ-Myc_10_ | 1 | 80 | — | — | 19 | 1 |
| Aβ-Mor_1_ | 46 | 38 | 39 | 2 | 16 | 6 |
| Aβ-Mor_2_ | 17 | 61 | 3 | 5 | 22 | 9 |
| Aβ-Mor_6_ | 3 | 70 | 1 | 1 | 26 | 1 |
| Aβ-Mor_10_ | 3 | 75 | 0 | 0 | 22 | 2 |
| Aβ- Flv_1_ | 33 | 42 | 22 | 6 | 25 | 5 |
| Aβ- Flv_2_ | 37 | 42 | 32 | 1 | 21 | 4 |
| Aβ- Flv_6_ | 21 | 58 | 18 | 1 | 21 | 3 |
| Aβ- Flv_10_ | 11 | 72 | 10 | 1 | 17 | 0 |

**^a^** Structure = A-Helix + B-Sheet + B-Bridge + Turn
